# Supplementary material for: Substantially enhanced homogeneous plastic flow in hierarchically nanodomained amorphous alloys
Source: Nat Commun. 2023 Jun 20;14:3670. doi: 10.1038/s41467-023-39296-6 (PMC10282017; doi:10.1038/s41467-023-39296-6)
Supplement: Supplementary file 1 — Supplementary Information [file 41467_2023_39296_MOESM1_ESM.pdf]

Supplementary Information for

Substantially enhanced homogeneous plastic flow in hierarchically nanodomained amorphous alloys

Ge Wu[1*], Sida Liu[2], Qing Wang[3], Jing Rao[4], Wenzhen Xia[5], Yong-Qiang Yan[1], Jürgen Eckert[6, 7], Chang Liu[8*], En Ma[8], Zhi-Wei Shan[1]

1. *Center for Advancing Materials Performance from the Nanoscale (CAMP-Nano) and Hysitron Applied Research Center in China (HARCC), State Key Laboratory for Mechanical Behavior of Materials, Xi'an Jiaotong University, 710049 Xi'an, China*
2. *Institute for Advanced Technology, Shandong University, Jinan, 250061, China*
3. *Laboratory for Microstructures, Institute of Materials, Shanghai University, Shanghai 200072, China*
4. *Max-Planck-Institut für Eisenforschung, Max-Planck-Straße 1, Düsseldorf 40237, Germany*
5. *School of Metallurgical Engineering, Anhui University of Technology, Maanshan 243000, China*
6. *Erich Schmid Institute of Materials Science, Austrian Academy of Sciences, Jahnstraße 12, Leoben A-8700, Austria*
7. *Department of Materials Science, Chair of Materials Physics, Montanuniversität Leoben, Jahnstraße 12, Leoben A-8700, Austria*
8. *Center for Alloy Innovation and Design (CAID), State Key Laboratory for Mechanical Behavior of Materials, Xi'an Jiaotong University, 710049 Xi'an, China*

Email: chang.liu@xjtu.edu.cn (C. Liu); gewuxjtu@xjtu.edu.cn (G. Wu)

Supplementary Table 1 | The values of mixing enthalpy[1] (kJ/mol) calculated by Miedema's model for atomic pairs of frequently-used metallic elements. The mixing enthalpy with small positive values (5-20 kJ/mol) are highlighted in green color.

	Mg	Al	Ca	Ti	Fe	Co	Ni	Cu	Zn	Zr	Pd	Ag	Ta	W	Ir	Pt	Au
Au	-32	-22	-60	-47	8	7	7	-9	-16	-74	0	-6	-32	12	13	4	
Pt	-35	-44	-55	-74	-13	-7	-5	-12	-29	-100	2	-1	-66	-20	0		
Ir	-13	-30	-23	-57	-9	-3	-2	0	-13	-76	6	16	-52	-16			
W	38	-2	57	-6	0	-1	-3	22	15	-9	-6	43	-7				
Ta	30	-19	60	1	-15	-24	-29	2	-3	3	-52	15					
Ag	-10	-4	-28	-2	28	19	15	2	-4	-20	-7						
Pd	-40	-46	-63	-65	-4	-1	0	-14	-33	-91							
Zr	6	-44	37	0	-25	-41	-49	-23	-29								
Zn	-4	1	-22	-15	4	-5	-9	1									
Cu	-3	-1	-13	-9	13	6	4										
Ni	-4	-22	-7	-35	-2	0											
Co	3	-19	2	-28	-1												
Fe	18	-11	25	-17													
Ti	16	-30	43														
Ca	-6	-20															
Al	-2																
Mg																	

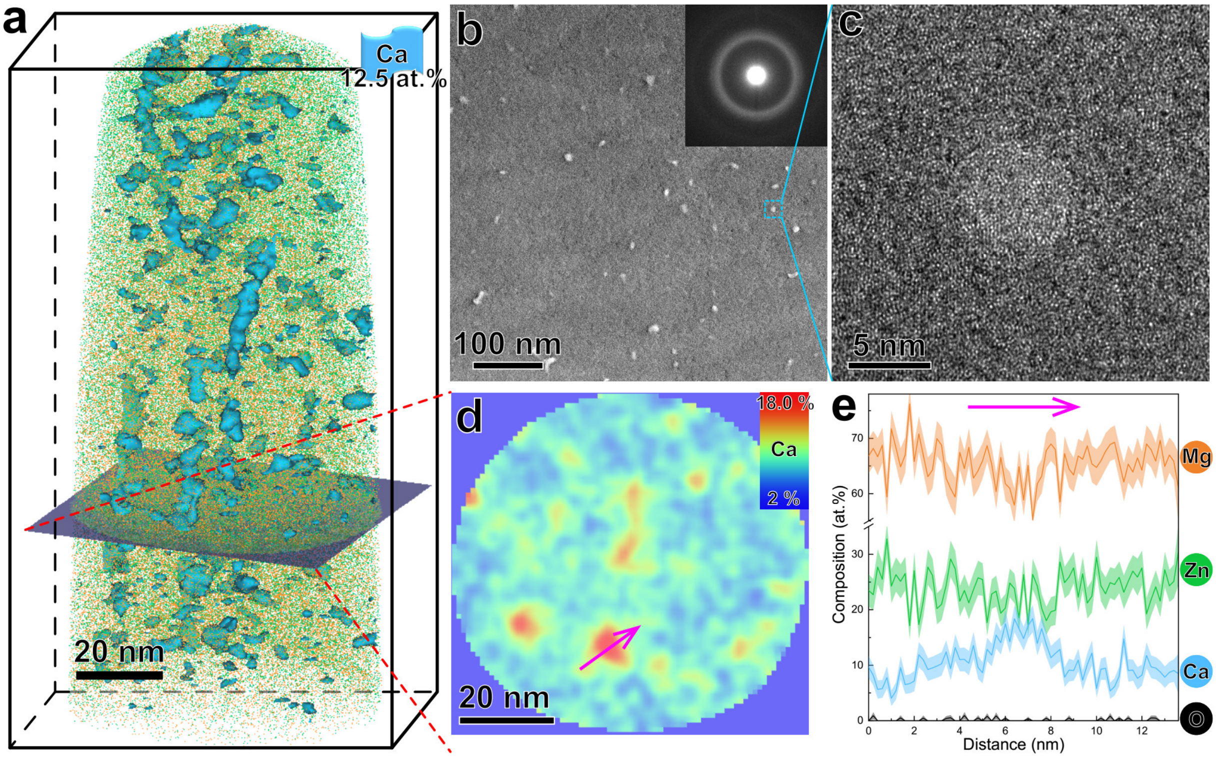

Supplementary Fig. 1 | Structure and composition of the $Mg_{64.6}Zn_{26.2}Ca_{8.8}O_{0.4}$ (at.%) reference amorphous alloy. a, 3D reconstruction of an APT dataset, showing that Ca enriched nano-filaments are embedded in the matrix. The Ca enriched regions are highlighted by an iso-concentration surface in terms of 12.5 at.% Ca. **b,** Plan-view TEM image, showing a dual-phase structure. The halo ring feature of the SAED pattern in the inset indicates an amorphous structure. **c,** HRTEM image of the corresponding region in (**b**), showing maze-like pattern of the amorphous domains. **d,** 2D contour plot in terms of the Ca concentration of a 1 nm-thick plan-view slice from (**a**), showing the distribution of the Ca-enriched amorphous domain. **e,** 1D compositional profile across the region indicated by the arrow in (**d**). The light shadows indicate statistical errors in terms of the standard deviations.

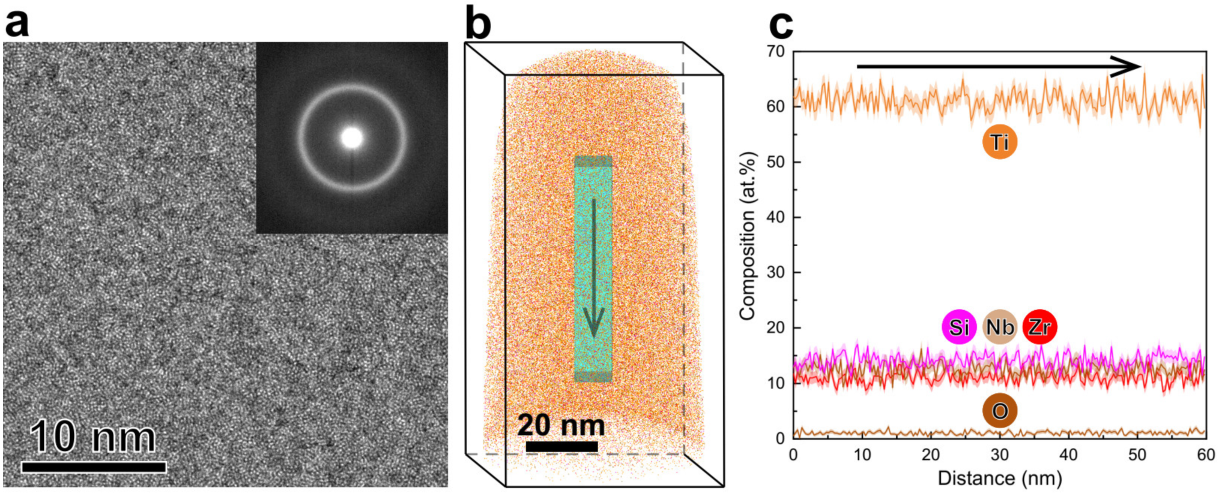

Supplementary Fig. 2 | Structure and composition of the $Ti_{60.7}Zr_{11.2}Nb_{12.6}Si_{14.4}O_{1.1}$ (at.%) reference amorphous alloy. a, Typical HRTEM image (main panel) and SAED pattern (inset), showing an amorphous structure. **b,** 3D reconstruction of an APT dataset. **c,** 1D compositional profile across the region indicated by a cuboid in (**b**), revealing the homogeneous distribution of Ti, Zr, Nb, Si and O.

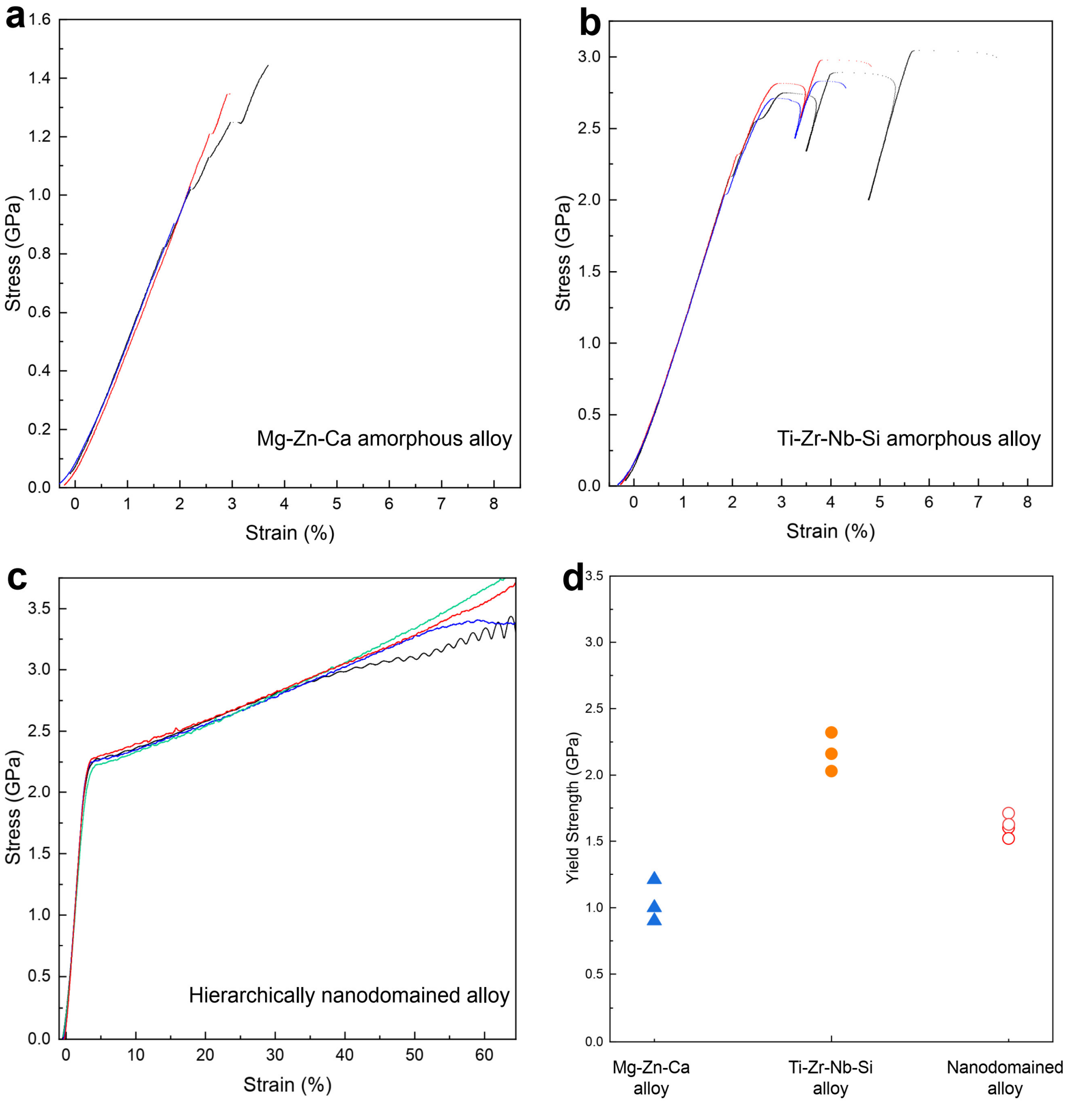

Supplementary Fig. 3 | Mechanical properties of the hierarchically nanodomained amorphous alloy, Mg-Zn-Ca amorphous alloy and Ti-Zr-Nb-Si amorphous alloy. a-c, Stress-strain curves of the alloys. The pillar compression tests under identical conditions were repeated for at least 3 times. **d,** Yield strength of the alloys.

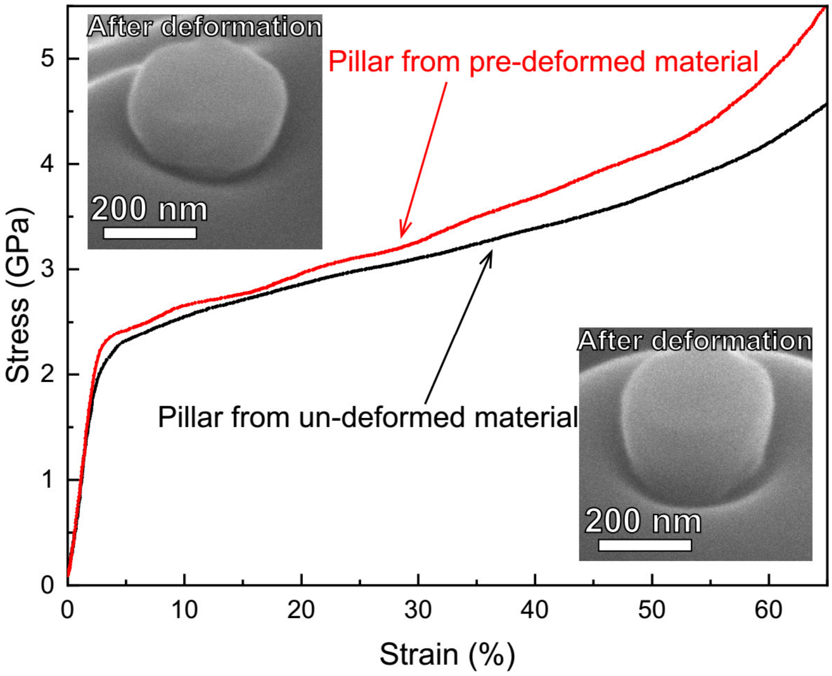

Supplementary Fig. 4 | Yield strength increase of the hierarchically nanodomained amorphous alloy after deformation. Compressive engineering stress-strain curves of the nano-pillars fabricated from pre-deformed and un-deformed materials. The nano-pillars have an identical dimension of 170 nm-diameter and 400 nm-length. The pre-deformed material was prepared by micro-indentation using a 3 μm-radius spherical diamond tip, to a depth of ~1 μm (Supplementary Fig. 5). The insets are SEM images of the deformed nano-pillars after deformation. Therefore, the compressed nano-pillar with the raw sample fabricated from the deformed material (top left inset) has an engineering strain of >70%. The nanopillar from the deformed sample also undergoes homogeneous plastic deformation. The diameter of the as-prepared nanopillars is 170 nm, close to the critical size (100 nm) for brittle to ductile transition in MGs[2,3]. Therefore, the homogeneous deformation of the nanopillar samples may be due to small size induced plasticity, but the 1 μm-diameter samples shown in Fig. 2a-d are free of the sample size effect.

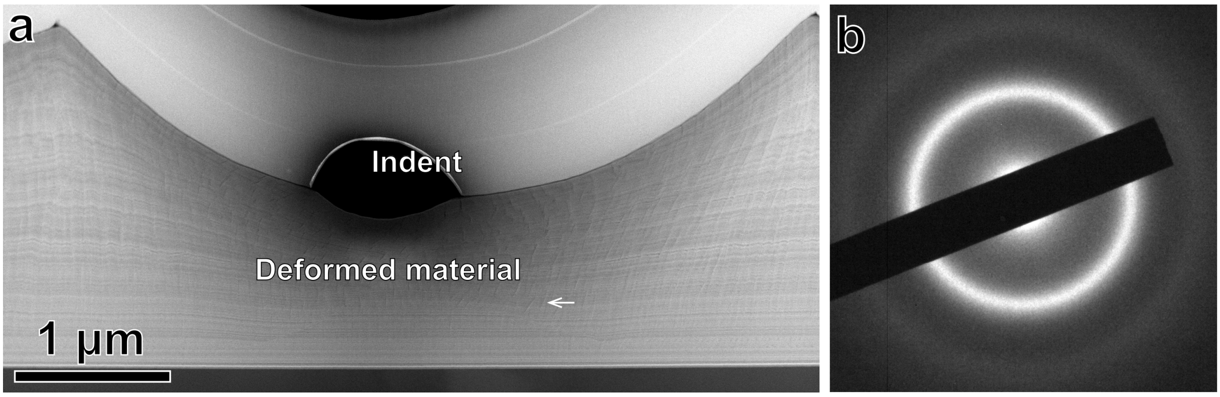

Supplementary Fig. 5 | Structure of deformed material beneath an indent on the hierarchically nanodomained amorphous alloy. a, HAADF-STEM image. The indentation was performed using a 3 μm-radius spherical diamond tip. The arrow denote enlarged STEM and EDS probing region shown in Fig. 3b and f. **b,** SAED pattern of the deformed regions, showing a broad halo ring.

Supplementary References

1 Takeuchi, A. & Inoue, A. Classification of bulk metallic glasses by atomic size difference, heat of mixing and period of constituent elements and its application to characterization of the main alloying element. *Mater. Trans.* **46**, 2817-2829 (2005).

2 Guo, H. *et al.* Tensile ductility and necking of metallic glass. *Nat. Mater.* **6**, 735-739 (2007).

3 Jang, D. & Greer, J. R. Transition from a strong-yet-brittle to a stronger-and-ductile state by size reduction of metallic glasses. *Nat. Mater.* **9**, 215-219 (2010).
